# Dynamic changes in intrinsic subtype, immunity status, and risk score before and after neoadjuvant chemo- and HER2-targeted therapy without pCR in HER2-positive breast cancers: A cross-sectional analysis

**DOI:** 10.1097/MD.0000000000029877

**Published:** 2022-08-05

**Authors:** Peixian Chen, Xiaofan Mao, Na Ma, Chuan Wang, Guangyu Yao, Guolin Ye, Dan Zhou

**Affiliations:** a Department of Breast Surgery, The First People’s Hospital of Foshan, Guangdong, China; b Clinical Research Institute, The First People’s Hospital of Foshan, Guangdong, China; c Department of Pathology, The First People’s Hospital of Foshan, Guangdong, China; d The First People’s Hospital of Foshan, Guangdong, China; e Breast Center, Department of General Surgery, Nanfang Hospital, Southern Medical University, Province, hina.

**Keywords:** biomarkers, breast cancer, HER2, neoadjuvant, PAM50

## Abstract

Very few studies have been done in HER2 positive patients without complete pathological response (pCR) after combined neoadjuvant chemo- and HER2-target therapy to investigate changes in intrinsic subtype, risk of recurrence (ROR) score, and immunity status before and after treatment.

Patients with nonmetastatic HER2-positive breast cancer failed to achieve pCR after neoadjuvant chemotherapy plus trastuzumab were included in current study. We examined the distribution of PAM50 subtypes, ROR score and immunity score in 25 paired baseline and surgical samples. The Miller–Payne grading system was used to evaluate the efficacy of the neoadjuvant therapy. It was observed that the distribution of intrinsic subtype, ROR category and immunity subgroup varied according to hormone receptor (HR) status. HER2-enriched and basal-like subtypes, median-high ROR categories and immunity-weak subgroup were dominant in baseline tumors. Compared to baseline samples, conversion of intrinsic subtype, ROR categories and immunity subgroups were found in 15 (60.0%), 13(52.0%), and 11(44.0%) surgical samples, respectively. The PAM50 subtype, ROR category, and immunity subgroup were concordant between baseline and surgical samples where nonluminal subtypes, median-high ROR categories and i-weak subgroup were still common.

In conclusion, the HER2-positive breast cancer is highly heterogeneous with a distribution of 72-gene expression varying according to HR co-expression. The dynamics of the 72-gene expression pre- and posttreatment may become novel biomarker for guiding adjuvant therapy and hence warrant further investigation.

## 1. Introduction

Approximately 15% to 25% of breast cancers (BCs) overexpress human epidermal growth factor 2 (HER2), which are historically aggressive, associated with poor prognosis and have a higher risk of relapse than HER2-negative cancers.^[1–4]^ The humanized anti-HER2 monoclonal antibody trastuzumab was the first molecularly targeted agent to be approved by the FDA for the treatment of patients with HER2-positive BCs. The addition of trastuzumab to chemotherapy has dramatically improved the outcomes in patients with HER2-positive breast cancer.^[5,6]^ In the adjuvant setting, a joint analysis of the North Central Cancer Treatment Group (NCCTG) N9831 trial and the National Adjuvant Breast and Bowel Project (NSABP) B-31 showed that trastuzumab plus chemotherapy resulted in a 37% improvement in the overall survival (OS) and a 40% improvement in disease-free survival.^[7]^ While data for neoadjuvant trastuzumab showed almost a doubled rate of pathologic complete response (pCR).^[8,9]^The patients who achieved pCR after neoadjuvant treatment have a better long-term outcome.^[10]^ In a meta-analysis of 36 trials enrolling 5800 patients with HER2-positive BCs receiving neoadjuvant therapy, those who obtained a pCR experienced superior event-free survival and OS compared with those who did not.^[11]^ Consequently, increasing the rate of pCR became the endpoint of neoadjuvant trials with the expectation of translation into improved survival. However, recurrences or metastases still occur in some patients with HER2-positive BCs, commonly seen in those presenting a non-pCR after neoadjuvant therapy, which constitutes a serious clinical problem. As a result, it is important to identify biomarkers suggesting HER2-positive breast tumors with poor responsiveness to neoadjuvant chemotherapy (NAC) and trastuzumab.

Gene expression profiles of primary tumors are highly predictive of distant metastasis in breast cancer.^[12,13]^ The expression signatures of the primary tumor are prognostic and to predict a patient’s outcome. The most thoroughly characterized classifier of molecular heterogeneity is the PAM50 signature, which uses the expression of 50 genes to stratify breast tumors into 4 major classes: basal-like (basal), HER2-enriched (Her2), luminal A (LumA), and luminal B (LumB).^[14,15]^ Numerous studies have demonstrated that the PAM50 subtype is a biomarker in the neoadjuvant setting^[16–18]^ and may even have a prognostic role for HER2-positive BC.^[19]^ In addition, immune signatures such as tumor-infiltrating lymphocytes and PD-L1 have been showed to add significance in predicting pCR beyond PAM50 intrinsic subtypes in HER2 positive BC.^[18,20]^ Therefore, the combination of PAM50 intrinsic subtype and immune signatures may further improve the accuracy of response evaluation to NAC in HER2-positive BC. The objective of this study was to use a 72-gene panel,^[21]^ which contains the 17 immunity genes, 19 proliferation genes, 11 Basal genes, 14 ER genes, 3 HER2E genes, 2 invasion genes, and 6 housekeeper genes, to assess their distribution and association with the efficacy of neoadjuvant chemotherapy plus trastuzumab of patients with nonmetastatic HER2-positive breast cancer who failed to achieved pCR after neoadjuvant therapy.

## 2. Methods

This study is reported as per the Strengthening the Reporting of Observational Studies in Epidemiology (STROBE) guideline (S1 STROBE Checklist).

### 2.1. Study population

This study included 25 nonmetastatic BC patients who received anthracycline and taxane plus trastuzumab-based neoadjuvant therapy followed by surgery at The First People’s Hospital of Foshan during January 2016 and December 2018. Patients received eight 21-day cycles of epirubicin (90 mg/m^2^, intravenously [i.v.] on day 1), cyclophosphamide (600 mg/m^2^, i.v. on day 1) following docetaxel (80–100 mg/m^2^, i.v. on day 1), trastuzumab (8 mg/kg, i.v. cycle 5 on day 1, followed by 6 mg/kg cycle 6 on day 1 to cycle 8 on day 1) or six 21-day cycles of docetaxel (75 mg/m^2^, i.v. on day 1), carboplatin (AUC = 5, i.v. on day 1), trastuzumab (8 mg/kg, i.v. cycle 1 on day 1, followed by 6 mg/kg cycle 2 on day 1 to cycle 6 on day 1) for NAC. We obtained biopsy and surgical samples of each patient, as a result, 50 breast tumor FFPE blocks were included for analysis.

According to American Society of Clinical Oncology/College of American Pathologists guidelines,^[22,23]^ pathologic HER2 positivity was defined as a score of 3+ by immunohistochemistry (IHC) staining or HER2/neu gene amplification by fluorescence in situ hybridization; tumors were defined as hormone receptor-positive by a finding of a least 1% of positive cells for estrogen receptor, progesterone receptor, or both, evaluated by IHC analysis.

### 2.2. Response assessment

According to the Miller–Payne (MP) grading system,^[24]^ we evaluated the efficacy of NAC by comparing the surgical specimens after NAC with the initial core biopsies. Each of the biopsy samples and surgical specimens was evaluated by 2 pathologists. The MP grading system is a 5-level classification method. Grades 1 to 4 are categorized as a partial pathological response and grade 5 is a complete pathological response. This study focused on patients without pCR (i.e., Grades 1–4) after NAC.

### 2.3. PAM50 subtyping

The PAM50 analyses were performed on archival paraffin-embedded tumor tissue. Hematoxylin and eosin stained sections were evaluated for the relevant area that met the following criteria: ≥10% viable tumor cells as well as <20% DCIS and/or nonneoplastic tissue. When a whole section could not meet the given criteria, a suitable area was marked for macrodissection. Then the area of relevance was measured, enabling deduction of the amount of unstained 10 µm sections needed. Deparaffinization, RNA purification, and PAM50 analysis were performed as described by the manufacturer, using the protocol for Prosigna (NanoString Technologies, Seattle), including use of the nCounter Flex system instrument and software.

### 2.4. Calculation of risk of relapse and proliferation scores

The subtype risk model was trained with a multivariable Cox model^[15]^ using the node-negative, untreated subset of the van de Vijver cohort.^[25]^ The risk of relapse (ROR) score was assigned to each test case using correlation to each subtype and proliferation score: ROR score = 0.01 × Basal + 0.07 + Her2 + 0.10 × LumA + 0.05 × LumB + 0.34 × Proliferation score. Proliferation score was mean of gene expression levels of all eleven proliferation genes (CCNB1, UBE2C, BIRC5, KNTC2, CDC20, PTTG1, RRM2, MKI67, TYMS, CEP55, CDCA1). A new sample’s gene expression profile was processed using a single sample predictor^[26]^ and normalized using Combat.^[27]^

### 2.5. Calculation of immunity scores

A compact of 17 immunity gene signature was generated containing APOBEC3G, CCL5, CCR2, CD2, CD27, CD3D, CD52, CORO1A, CXCL9, GZMA, GZMK, HLA-DMA, IL2RG, LCK, PRKCB, PTPRC, and SH2D1A after microarray data mining by Yang et al.^[21]^ An Immunity score was calculated by averaging gene expression values of 17 immunity genes as “unscaled immunity score” and then scaled between 0 and 100 for each sample using the formula: 30× (unscaled immunity score+1.4). For Immunity score group classification, the patients were divided into 2 groups, “immunity-weak (i-weak)” and “immunity-strong (i-strong),” based on their Immunity score values using the cutoff value of 42 that was derived from the combined data using X-tile.^[27]^

### 2.6. Statistical Analysis

The small sample size makes it difficult for a meaningful statistical result, therefore data are only shown in a descriptive way.

### 2.7. Ethics approval and consent to participate

The study was conducted in accordance with the Declaration of Helsinki and was approved by the Institutional Review Board of The First People’s Hospital of Foshan, approval number [L2021-9]. Written informed consent was waived by the Institutional Review Board of The First People’s Hospital of Foshan.

## 3. Results

### 3.1. Clinicopathological characteristics at baseline

Patient characteristics are summarized in Table 1. The mean age of patients was 48 years (range: 28–61 years). Most patients were premenopausal (n = 17;68%), absent of family history of breast cancer (n = 22;88%) and had clinically node-positive disease (n = 17; 68%), T2 tumors (n = 17; 68%), or histologically grade II tumors (n = 17; 68%). All tumors in this study were histologically diagnosed as invasive ductal carcinomas. Approximately half of the patients had hormone receptor (HR)-negative disease (n = 13; 52%). Baseline clinicopathological variables were comparable between HR-negative and HR-positive disease.

**Table 1:** Baseline clinicopathological characteristics.

Variables	N	HR subtypes at baseline	
		HR-negative (n = 13)	HR-positive(n = 12)
Age, median (range), y	48 (28–61)	49 (32–61)	46.5 (28–61)
Menstrual status, n (%)			
Premenopause	17 (68%)	9 (69%)	8 (67%)
Postmenopause	8 (32%)	4 (31%)	4 (33%)
Family history of breast cancer, n (%)			
Yes	3 (12%)	2 (15%)	1 (8%)
No	22 (88%)	11 (85%)	11 (92%)
Tumor size, median (cm) and range	3.5 (2–6.5)	3.8 (2–6.5)	3.0 (2–4.8)
cN			
cN0	8 (32%)	4 (31%)	4 (33%)
cN1	16 (64%)	8 (62%)	8 (67%)
cN2-3	1 (4%)	1 (8%)	0
cTNM stage, n (%)			
I	2 (8%)	1 (8%)	1 (8%)
II	20 (80%)	9 (69%)	11 (92%)
III	3 (12%)	3 (23%)	0 (0)
Histologic grade on biopsy, n (%)			
2	17 (68%)	8 (62%)	9 (75%)
3	8 (32%)	5 (38%)	3 (25%)
Ki-67 index on biopsy (median and range)	30 (15–90)	30 (20–40)	40 (15–90)
MP grading, n (%)			
MP1-2	6 (24.0)	4 (30.8)	2 (16.7)
MP3-4	19 (76.0)	9 (69.2)	10 (83.3)

### 3.2. Intrinsic subtype identification at baseline

The majority of tumor samples were identified as HER2-enriched (Her2) (n = 10; 40%) and basal-like (Basal) (n = 8;32%), followed by luminal A (LumA) (n = 4;16%) and luminal B (LumB) (n = 3;12%) based on PAM50 subtype. Two thirds of tumor samples were classified into intermedia-risk group (68.0%) and approximately one third were categorized into high-risk group (28.0%) according to ROR scores. The majority of tumor samples were identified as i-weak (n = 18; 72%). Of note, no LumB tumor was identified in HR-negative disease, and neither was low ROR risk category in HR-positive disease. Furthermore, the HER2-enriched subtype was identified in 46.2% (n = 6) and 33.3% (n = 4) of HR-negative and HR-positive disease, respectively (Table 2).

**Table 2:** The distribution of subgroups in baseline tumors and surgical specimens.

	Baseline tumors (N = 25)			Surgical specimen (N = 25)
	Total, n (%)	HR-positive (n = 12)	HR-negative (n = 13)	
PAM50 subtype				
Luminal A	4 (16.0)	2 (16.7)	2 (15.4)	11 (44.0)
Luminal B	3 (12.0)	3 (25.0)	0	0
HER2-enriched	10 (40.0)	4 (33.3)	6 (46.2)	5 (20.0)
Basal-like	8 (32.0)	3 (25.0)	5 (38.5)	9 (36.0)
ROR classification				
Low risk	1 (4.0)	0	1 (7.7)	7 (28.0)
Intermedia risk	17 (68.0)	10 (83.3)	7 (53.8)	14 (56.0)
High risk	7 (28.0)	2 (16.7)	5 (38.5)	4 (16.0)
Immunity subgroup				
I-strong	7 (28.0)	2 (16.7)	5 (28.5)	10 (40.0)
I-weak	18 (72.0)	10 (83.3)	8 (61.5)	15 (60.0)

### 3.3. Intrinsic subtype identification at surgery

The majority of surgical samples were identified as LumA (n = 11; 44%), followed by Basal (n = 9; 36%) and Her2 (n = 5; 20%). No LumB subtype was found at surgery. Intermedia-risk ROR category (n = 14; 56%) and i-weak group (n = 15; 60%) were most common among surgical samples (Table 2).

### 3.4. Treatment activity

Since this study focused on those who did not achieve a pCR after neoadjuvant therapy, response following NAC based on MP classification ranged from Grades 1 to 4. MP Grades 1 to 2 and Grades 3 to 4 occurred in 6 (24%) and 19 (76%) patients, respectively.

### 3.5. PAM50 subtype conversion between baseline and surgery

**Figure 1.**
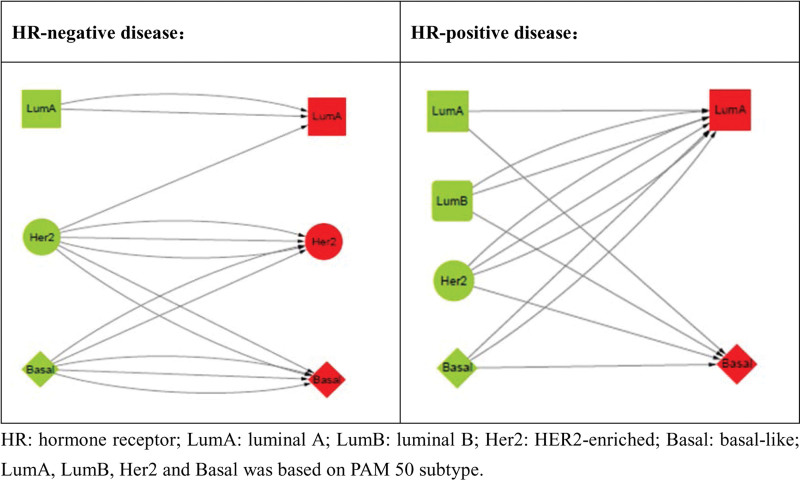
Intrinsic subtype conversion between baseline and surgery.

Compared to baseline samples, subtype alteration was found in 15 surgical specimens, 5 in HR-negative disease and 10 in HR-positive disease, respectively. In HR-negative group, the luminal subtypes at baseline remained the same in surgical specimens and baseline nonluminal subtype converting to luminal subtype at surgery just happened in one case. In HR-positive group, luminal subtype at baseline to nonluminal subtype at surgery was found in 2 cases and nonluminal subtype at baseline to luminal subtype at surgery was noticed in 5 cases (Fig. 1).

### 3.6. ROR category conversion between baseline and surgery

**Figure 2.**
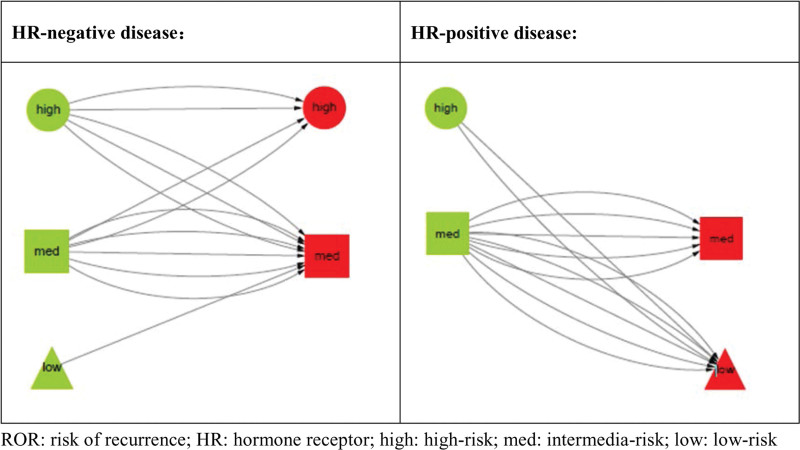
ROR category conversion between baseline and surgery. ROR = risk of recurrence.

ROR category changes happened in 13 surgical specimens compared with baseline samples, 6 in HR-negative disease and 7 in HR-positive disease, respectively. In HR-negative group, risk downgrading [(high risk to intermedia risk (n = 3)] and risk upgrading [intermedia risk to high risk (n = 2) and low risk to intermedia risk (n = 1)] between baseline and surgery took place in 3 and 3 cases, respectively. In HR-positive group, no risk upgrading after neoadjuvant therapy was found. Risk downgrading between baseline and surgery were identified in 7 cases with 2 of high risk to low risk and 5 of intermedia risk to low risk (Fig. 2).

### 3.7. Immunity group conversion between baseline and surgery

**Figure 3.**
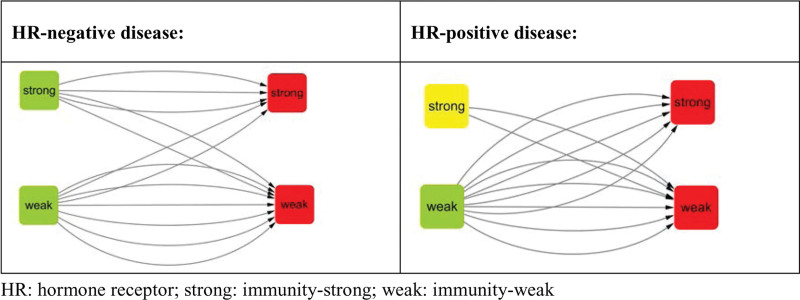
Immunity group conversion between baseline and surgery.

Immunity group changes took place in 11 surgical specimens with 4 in HR-negative and 7 in HR-positive disease. In HR-negative disease, conversion between baseline and surgery appeared in 4 cases with 2 of i-strong to i-weak and 2 of i-weak to i-strong. In HR-positive disease, increase and reduction in immunity scores were found in 5 and 2 cases, respectively (Fig. 3).

### 3.8. Follow-up

All patients were alive with a median follow-up time of 39.0 months (range: 28.2–58.8 months). In the meantime, recurrence events occurred in 3 patients: one with local recurrence and the other 2 with distant recurrence. All these patients had nonluminal subtype, high recurrence risk and weakly expressed immunity-related genes at baseline (Table 3).

**Table 3:** Characteristics of patients with recurrent events.

Variables	Patient A	Patient B	Patient C
Age, y	47	43	51
Menstrual status	Premenopause	Premenopause	Premenopause
Family history of breast cancer	no	No	yes
Baseline tumor size, cm	4	6.5	4.2
cN	cN1	cN3	cN0
cTNM stage	II	III	II
Histologic grade on biopsy	2	2	2
HR status	HR-negative	HR-negative	HR-positive
Ki-67 index on biopsy	25	40	40
Recurrent event	Brain metastasis	Extensive metastasis	Recurrence on ipsilateral chest wall
MP classification	4	4	1
Baseline PAM50 subtype	Basal-like	Basal-like	HER2-enriched
Baseline ROR category	Intermedia risk	High risk	High risk
Baseline immunity subgroup	i-weak	i-weak	i-weak
Posttreatment PAM50 subtype	Basal-like	Basal-like	Luminal A
Posttreatment ROR category	High risk	High risk	Low risk
Posttreatment immunity subgroup	i-weak	i-weak	i-strong

## 4. Discussion

Neoadjuvant chemotherapy with addition of anti-Human Epidermal Growth Factor Receptor 2 therapy is a standard of care for early HER2-positive breast cancer. The association of sensitivity to NAC in terms of pCR achievement with better long-term outcomes has been proved, therefore identifying biomarkers that predict pCR has gained growing interest. Among the pCR predictors that are currently in use, PAM50-defined molecular subtypes stand out as valuable tools to stratify patients based on expected pCR achievement.^[28]^ In this study, we used a 72-gene panel including PAM50 genes to examine the effectiveness of NAC in HER2-positive disease. We have focused on those who failed to achieve pCR after NAC plus trastuzumab.

Consistent with other literature,^[16,19]^ our study supports that HER2-positive disease is biologically heterogenous with a distribution of the intrinsic subtypes and gene expression varying according to HR co-expression. The HER2-enriched subtype was most commonly identified regardless of the HR status in baseline samples. In fact, numerous studies have established the role of PAM50 intrinsic subtype as a biomarker for HER2-positive BC in the neoadjuvant setting.^[29–31]^ HER2-enriched and basal-like subtypes have been reported to be strong predictors of pCR in both single and dual anti-HER2 therapy groups.^[32–34]^ However, some patients may fail to achieve pCR and those with residual disease at surgery stand a higher ROR and death,^[35]^ so it is important to look into the residual disease for why and what to do.

In this study, we also examined the subtype distribution and gene expression in residual tumor samples treated with NAC + trastuzumab. It is noteworthy that nonluminal subtypes (HER2-enriched or basal-like) and high-or median-risk of recurrence category were identified in more than half of the residual tumors in this study. An analysis of comparing PAM50 subtypes in 123 paired primary and metastatic tissues revealed that the rate of subtype conversion was 0% in basal-like tumors, 23.1% in HER2-enriched tumors, 30% in luminal B tumors and 55.3% in luminal A tumors, and subtype concordance was high for basal-like (100%), HER2-E (76.9%), and luminal B (70.0%) tumors.^[36]^ Another analysis of comparing IHC subtypes in 119 paired primary and metastatic tissues disclosed that tumor phenotype discordance was associated with worse postrecurrence and overall survival and those who turned into triple-negative experienced the poorest outcome.^[37]^ In our study, the rate of subtype conversion was 50% in basal-like tumors, 60% in HER2-enriched tumors, 100% in luminal B tumors and 25% in luminal A tumors after NAC; however, cases changing into nonluminal subtypes outnumbered those into luminal subtype. Basal-like subtypes were identified at baseline and maintained after NAC in 2 recurrent cases. Therefore, we assume that failure of conversion into luminal subtype and of risk of recurrence downgrading after therapy were the possible reasons for poor response to NAC plus trastuzumab. The KATHERINE trial indicated that for those with residual tumors ≥0.5 cm after neoadjuvant therapy, the substitution of T-DM1 for trastuzumab reduced both the risk of invasive disease recurrence and distant recurrence.^[38]^ We think that the alteration of PAM50 subtype and ROR category before and after neoadjuvant therapy may be a novel indicator for guiding adjuvant therapy, for those showing nonluminal subtype or median-high risk of recurrence in residual disease may require escalated adjuvant therapy. Currently, the studies investigating the association of outcome and the PAM50 subtype conversion pre- and post-NAC are rare. Further studies are required in this field.

In addition, we examined the association of immunity-related genes expression with the sensitivity to NAC. Increasing evidence suggest that the immune cells in the tumor microenvironment can modulate tumor response to chemotherapy and anti-HER2 targeted therapies.^[39,40]^ Higher tumor-infiltraing lymphocytes (TILs) have been associated with both increased likelihood of pCR following NAC^[41]^ and with improved prognosis^[42]^ in early HER2-positive BC. An analysis of single cell RNA-seq data by Yang et al^[21]^ indicated that expression levels of immunity genes might reflect the number of TILs. In our study, most of breast cancer samples were stratified into the immunity-weak group, suggesting that low levels of TILs could be a reason for poor response among our patients. In addition, it is noteworthy that enhanced expression of the immunity genes was found in 7 cases (2 in HR-negative group and 5 in HR- positive group), indicating that these patients might benefit from immunotherapy in the adjuvant setting. Yang et al^[21]^ also demonstrated that the immunity score could be a companion predictive marker in addition to PDL-1 expression for immune-checkpoint inhibitors. Immunotherapy based on PD-1 is emerging as a promising alternative or supplement to conventional chemotherapy in oncology. Nevertheless, nonresponders are not uncommon. A literature review supports that TILs mitochondrial biogenesis plays a pivotal role in response to anti-PD-1 therapies.^[43]^ Another in vitro experimental study showed that chemotherapy-exposed T cells might have lingering dysfunction in a way that the mitochondrial energy reserve was damaged.^[44]^ These findings reflected that TILs could be stratified into a number of distinct phenotypes according to varying mitochondrial levels. Based on this hypothesis, we speculate that the immunity gene expression of distinct TILs phenotypes (i.e., metabolically active T cells) may be a predictive factor for immunotherapy or escalated treatment for patients without pCR after NAC. Further study in this field is warranted.

There are several limitations in this study. First, the retrospective nature may cause misinterpretation because RNAs in specimens would degrade quickly as time gets by. Second, it may be difficult to achieve statistical significance due to small sample analysis. Third, we did not perform gene expression analysis, which limited the possibility to derive new gene signatures and identify new biological processes associated with treatment response. We will continue the study in a larger cohort prospectively using fresh specimens and perform RNA sequencing analysis.

## 5. Conclusions

In conclusion, the HER2-positive breast cancer is highly heterogeneous with a distribution of 72-gene expression varying according to HR co-expression. The dynamics of the 72-gene expression pre- and posttreatment may become novel biomarker for guiding adjuvant therapy and hence warrant further investigation.

## References

[1] Debiasi M Polanczyk CA Ziegelmann P. Efficacy of anti-HER2 agents in combination with adjuvant or neoadjuvant chemotherapy for early and locally advanced HER2-positive breast cancer patients: a network meta-analysis. Front Oncol. 2018;8:156. 29872641 10.3389/fonc.2018.00156 PMC5972314

[2] Curigliano G Viale G Bagnardi V. Clinical relevance of HER2 overexpression/amplification in patients with small tumor size and node-negative breast cancer. J Clin Oncol. 2009;27:5693–9. 19884553 10.1200/JCO.2009.22.0962

[3] Rouanet P Roger P Rousseau E. HER2 overexpression a major risk factor for recurrence in pT1a-bN0M0 breast cancer: results from a French regional cohort. Cancer Med. 2014;3:134–42. 24407937 10.1002/cam4.167 PMC3930398

[4] Ferretti G Felici A Papaldo P. HER2/neu role in breast cancer: from a prognostic foe to a predictive friend. Curr Opin Obstet Gynecol. 2007;19:56–62. 17218853 10.1097/GCO.0b013e328012980a

[5] Rimawi MF Schiff R Osborne CK. Targeting HER2 for the treatment of breast cancer. Annu Rev Med. 2015;66:111–28. 25587647 10.1146/annurev-med-042513-015127

[6] Gutierrez C Schiff R. HER2: biology, detection, and clinical implications. Arch Pathol Lab Med. 2011;135:55–62. 21204711 10.1043/2010-0454-RAR.1 PMC3242418

[7] Perez EA Romond EH Suman VJ. Trastuzumab plus adjuvant chemotherapy for human epidermal growth factor receptor 2-positive breast cancer: planned joint analysis of overall survival from NSABP B-31 and NCCTG N9831. J Clin Oncol. 2014;32:3744–52. 25332249 10.1200/JCO.2014.55.5730 PMC4226805

[8] Buzdar AU Ibrahim NK Francis D. Significantly higher pathologic complete remission rate after neoadjuvant therapy with trastuzumab, paclitaxel, and epirubicin chemotherapy: results of a randomized trial in human epidermal growth factor receptor 2-positive operable breast cancer. J Clin Oncol. 2005;23:3676–85. 15738535 10.1200/JCO.2005.07.032

[9] Sheikh F Nazir A Yasmeen S. Pathologic complete response in HER2-positive breast cancer patients receiving trastuzumab in neoadjuvant setting. Asia-Pacific J Clin Oncol. 2019;29:159–63. 10.29271/jcpsp.2019.02.159 30700356

[10] Gianni L Eiermann W Semiglazov V. Neoadjuvant and adjuvant trastuzumab in patients with HER2-positive locally advanced breast cancer (NOAH): follow-up of a randomised controlled superiority trial with a parallel HER2-negative cohort. Lancet Oncol. 2014;15:640–7. 24657003 10.1016/S1470-2045(14)70080-4

[11] Broglio KR Quintana M Foster M. Association of pathologic complete response to neoadjuvant therapy in HER2-positive breast cancer with long-term outcomes: a meta-analysis. JAMA Oncol. 2016;2:751–60. 26914222 10.1001/jamaoncol.2015.6113

[12] Ramaswamy S Ross KN Lander ES. A molecular signature of metastasis in primary solid tumors. Nat Genet. 2003;33:49–54. 12469122 10.1038/ng1060

[13] Paik S Shak S Tang G. A multigene assay to predict recurrence of tamoxifen-treated, node-negative breast cancer. N Engl J Med. 2004;351:2817–26. 15591335 10.1056/NEJMoa041588

[14] Perou CM Sorlie T Eisen MB. Molecular portraits of human breast tumours. Nature. 2000;406:747–52. 10963602 10.1038/35021093

[15] Parker JS Mullins M Cheang MC. Supervised risk predictor of breast cancer based on intrinsic subtypes. J Clin Oncol. 2009;27:1160–7. 19204204 10.1200/JCO.2008.18.1370 PMC2667820

[16] Gavila J Oliveira M Pascual T. Safety, activity, and molecular heterogeneity following neoadjuvant non-pegylated liposomal doxorubicin, paclitaxel, trastuzumab, and pertuzumab in HER2-positive breast cancer (Opti-HER HEART): an open-label, single-group, multicenter, phase 2 trial. BMC Med. 2019;17:8. 30621698 10.1186/s12916-018-1233-1 PMC6325829

[17] Takahashi Y Iwamoto T Suzuki Y. Evaluation of therapeutic target gene expression based on residual cancer burden classification after neoadjuvant chemotherapy for HER2-negative breast cancer. Clin Breast Cancer. 2020;20:117–124 e114. 31570267 10.1016/j.clbc.2019.07.001

[18] Dieci MV Prat A Tagliafico E. Integrated evaluation of PAM50 subtypes and immune modulation of pCR in HER2-positive breast cancer patients treated with chemotherapy and HER2-targeted agents in the CherLOB trial. Ann Oncol. 2016;27:1867–73. 27484801 10.1093/annonc/mdw262

[19] Dieci MV Miglietta F Griguolo G. Biomarkers for HER2-positive metastatic breast cancer: beyond hormone receptors. Cancer Treat Rev. 2020;88:102064. 32622272 10.1016/j.ctrv.2020.102064

[20] Miller LD Chou JA Black MA. Immunogenic subtypes of breast cancer delineated by gene classifiers of immune responsiveness. Cancer Immunol Res. 2016;4:600–10. 27197066 10.1158/2326-6066.CIR-15-0149 PMC4930674

[21] Yang B Chou J Tao Y. An assessment of prognostic immunity markers in breast cancer. npj Breast Cancer. 2018;4:35. 30393759 10.1038/s41523-018-0088-0 PMC6206135

[22] Hatzis C Pusztai L Valero V. A genomic predictor of response and survival following taxane-anthracycline chemotherapy for invasive breast cancer. JAMA. 2011;305:1873–81. 21558518 10.1001/jama.2011.593 PMC5638042

[23] Wolff AC Hammond ME Schwartz JN. American Society of Clinical Oncology/College of American Pathologists guideline recommendations for human epidermal growth factor receptor 2 testing in breast cancer. Arch Pathol Lab Med. 2007;131:18–43. 19548375 10.5858/2007-131-18-ASOCCO

[24] Ogston KN Miller ID Payne S. A new histological grading system to assess response of breast cancers to primary chemotherapy: prognostic significance and survival. Breast. 2003;12:320–7. 14659147 10.1016/s0960-9776(03)00106-1

[25] van de Vijver MJ He YD van’t Veer LJ. A gene-expression signature as a predictor of survival in breast cancer. N Engl J Med. 2002;347:1999–2009. 12490681 10.1056/NEJMoa021967

[26] Hu Z Fan C Livasy C. A compact VEGF signature associated with distant metastases and poor outcomes. BMC Med. 2009;7:9. 19291283 10.1186/1741-7015-7-9 PMC2671523

[27] Camp RL Dolled-Filhart M Rimm DL. X-tile: a new bio-informatics tool for biomarker assessment and outcome-based cut-point optimization. Clin Cancer Res. 2004;10:7252–9. 15534099 10.1158/1078-0432.CCR-04-0713

[28] Chica-Parrado MR Godoy-Ortiz A Jiménez B. Resistance to neoadjuvant treatment in breast cancer: clinicopathological and molecular predictors. Cancers. 2020;12:2012. 10.3390/cancers12082012 PMC7463925 32708049

[29] Llombart-Cussac A Cortés J Paré L. HER2-enriched subtype as a predictor of pathological complete response following trastuzumab and lapatinib without chemotherapy in early-stage HER2-positive breast cancer (PAMELA): an open-label, single-group, multicentre, phase 2 trial. Lancet Oncol. 2017;18:545–54. 28238593 10.1016/S1470-2045(17)30021-9

[30] Guarneri V Dieci MV Bisagni G. De-escalated therapy for HR+/HER2+ breast cancer patients with Ki67 response after 2-week letrozole: results of the PerELISA neoadjuvant study. Ann Oncol. 2019;30:921–6. 30778520 10.1093/annonc/mdz055 PMC6594455

[31] Prat A Pascual T De Angelis C. HER2-enriched subtype and ERBB2 expression in HER2-positive breast cancer treated with dual HER2 blockade. J Natl Cancer Inst. 2020;112:46–54. 31037288 10.1093/jnci/djz042 PMC7850037

[32] Prat A Bianchini G Thomas M. Research-based PAM50 subtype predictor identifies higher responses and improved survival outcomes in HER2-positive breast cancer in the NOAH study. Clin Cancer Res. 2014;20:511–21. 24443618 10.1158/1078-0432.CCR-13-0239

[33] Prat A Fan C Fernandez A. Response and survival of breast cancer intrinsic subtypes following multi-agent neoadjuvant chemotherapy. BMC Med. 2015;13:303. 26684470 10.1186/s12916-015-0540-z PMC4683815

[34] Diaz-Redondo T Lavado-Valenzuela R Jimenez B. Different pathological complete response rates according to PAM50 subtype in HER2+ breast cancer patients treated with neoadjuvant pertuzumab/trastuzumab vs. trastuzumab plus standard chemotherapy: an analysis of real-world data. Front Oncol. 2019;9:1178. 31750258 10.3389/fonc.2019.01178 PMC6848376

[35] Cortazar P Zhang L Untch M. Pathological complete response and long-term clinical benefit in breast cancer: the CTNeoBC pooled analysis. Lancet. 2014;384:164–72. 24529560 10.1016/S0140-6736(13)62422-8

[36] Cejalvo JM Martinez de Duenas E Galvan P. Intrinsic subtypes and gene expression profiles in primary and metastatic breast cancer. Cancer Res. 2017;77:2213–21. 28249905 10.1158/0008-5472.CAN-16-2717 PMC5822682

[37] Dieci M Barbieri E Piacentini F. Discordance in receptor status between primary and recurrent breast cancer has a prognostic impact: a single-institution analysis. Ann Oncol. 2013;24:101–8. 23002281 10.1093/annonc/mds248

[38] von Minckwitz G Huang CS Mano MS. Trastuzumab emtansine for residual invasive HER2-positive breast cancer. N Engl J Med. 2019;380:617–28. 30516102 10.1056/NEJMoa1814017

[39] Ferris RL Jaffee EM Ferrone S. Tumor antigen-targeted, monoclonal antibody-based immunotherapy: clinical response, cellular immunity, and immunoescape. J Clin Oncol. 2010;28:4390–9. 20697078 10.1200/JCO.2009.27.6360 PMC2954137

[40] Bianchini G Gianni L. The immune system and response to HER2-targeted treatment in breast cancer. Lancet Oncol. 2014;15:e58–68. 24480556 10.1016/S1470-2045(13)70477-7

[41] Denkert C von Minckwitz G Darb-Esfahani S. Tumour-infiltrating lymphocytes and prognosis in different subtypes of breast cancer: a pooled analysis of 3771 patients treated with neoadjuvant therapy. Lancet Oncol. 2018;19:40–50. 29233559 10.1016/S1470-2045(17)30904-X

[42] Dieci MV Conte P Bisagni G. Association of tumor-infiltrating lymphocytes with distant disease-free survival in the ShortHER randomized adjuvant trial for patients with early HER2+ breast cancer. Ann Oncol. 2019;30:418–23. 30657852 10.1093/annonc/mdz007 PMC6442655

[43] Akbari H Taghizadeh-Hesary F Bahadori M. Mitochondria determine response to anti-programmed cell death protein-1 (anti-PD-1) immunotherapy: an evidence-based hypothesis. Mitochondrion. 2022;62:151–8. 34890822 10.1016/j.mito.2021.12.001

[44] Das RK O’Connor RS Grupp SA. Lingering effects of chemotherapy on mature T cells impair proliferation. Blood Adv. 2020;4:4653–64. 33002133 10.1182/bloodadvances.2020001797 PMC7556159

